# Packer’s Products

**Published:** 1875-11

**Authors:** 


### PACKER'S PRODUCTS.

Many letters have reached us in commendation of the Tar-Soap of which we have before spoken. The doctors like it, and are now in the practice of using it in all kinds of sores and skin-diseases. Gentlemen have called upon us to express their gratification at its effect in relieving the pain and soreness of boils, felons, and painful swellings. They follow the directions and make a thick lather, with which they profusely plaster the inflamed parts, and allow it to dry there. The effect is well-nigh magical. The same is true of those annoying little pimples which disfigure so many faces, especially in the young. The action of this completely saponified tar, is to destroy the disease-germs, and to neutralize the poison which causes the eruption. Doubtless much of this power is due to the creosote, or empyreumatic oil, contained in the pine-tar; but we apprehend that its healing and soothing effect is due to its balsamic properties. Whatever property effects the relief and promotes the cure, we can only say that this tar-soap of Packer's has won a high place in our regard, not only as a delightful toilet preparation for daily use, of great value in cleansing and purifying the skin, of unexcelled power as a neutralizer of the bad odors induced by pent-up perspiration,—but also as a simple and valuable remedy for many skin-affections, and a perfect substitute for salves, ointments, and other less cleanly dressings.

The Packer Company have now introduced an article which Mr. Packer calls Packer's Charm. It is a solution of great fragrance, and evidently composed of soothing, emmollient substances. It is a beautifier of the skin, inasmuch as it softens all roughnesses and asperities, and heals irritations and eruptions, from whatever cause. It certainly "works to a charm," and is therefore rightly named. We believe it to be a useful preparation, and will speak of it again after further tests.

